# Quality assessment of patient-facing urologic telesurgery content using validated tools

**DOI:** 10.1007/s11701-025-02871-8

**Published:** 2025-10-14

**Authors:** Tarak Davuluri, Paul Gabriel, Matthew Wainstein, Obi Ekwenna

**Affiliations:** University of Toledo College of Medicine and Life Sciences, Toledo, USA

**Keywords:** AI chatbots, Urologic telesurgery, DISCERN, PEMAT

## Abstract

**Introduction:**

With increasing accessibility to Artificial Intelligence (AI) chatbots, the precision and clarity of medical information provided require rigorous assessment. Urologic telesurgery represents a complex concept that patients will investigate using AI. We compared ChatGPT and Google Gemini in providing patient-facing information on urologic telesurgical procedures.

**Methods:**

19 questions related to urologic telesurgery were generated using general information from the American Urologic Association (AUA) and European Robotic Urology Section (ERUS). Questions were organized into 4 categories (Prospective, Technical, Recovery, Other) and directly typed into ChatGPT 4o and Google Gemini 2.5 (non-paid versions). For each question, a new chat was started to prevent any continuation of answers. Three reviewers independently reviewed the responses using two validated healthcare tools: DISCERN (quality) and Patient Education Material Assessment Tool (understandability and actionability).

**Results:**

Mean DISCERN scores (out of 80) were higher for Gemini than ChatGPT in all domains except “Other”. Prospective 49.2 versus 39.1; technical 52.3 versus 44.3; recovery 53.7 versus 45.4; other 54.3 versus 56.5; overall 52.4 versus 45.8 (Fig. 1). PEMAT-*P* understandability uniformly exceeded 70% for both platforms: prospective 80.0% versus 71.7%; technical 80.1% versus 79.8%; recovery 79.2% versus 80.1%; other 79.2% versus 81.3%; overall 79.7% versus 78.1% (Fig. 2). Actionability was uniformly low; only Gemini met the 70% threshold in the prospective domain (Fig. 3).

**Fig. 1:**
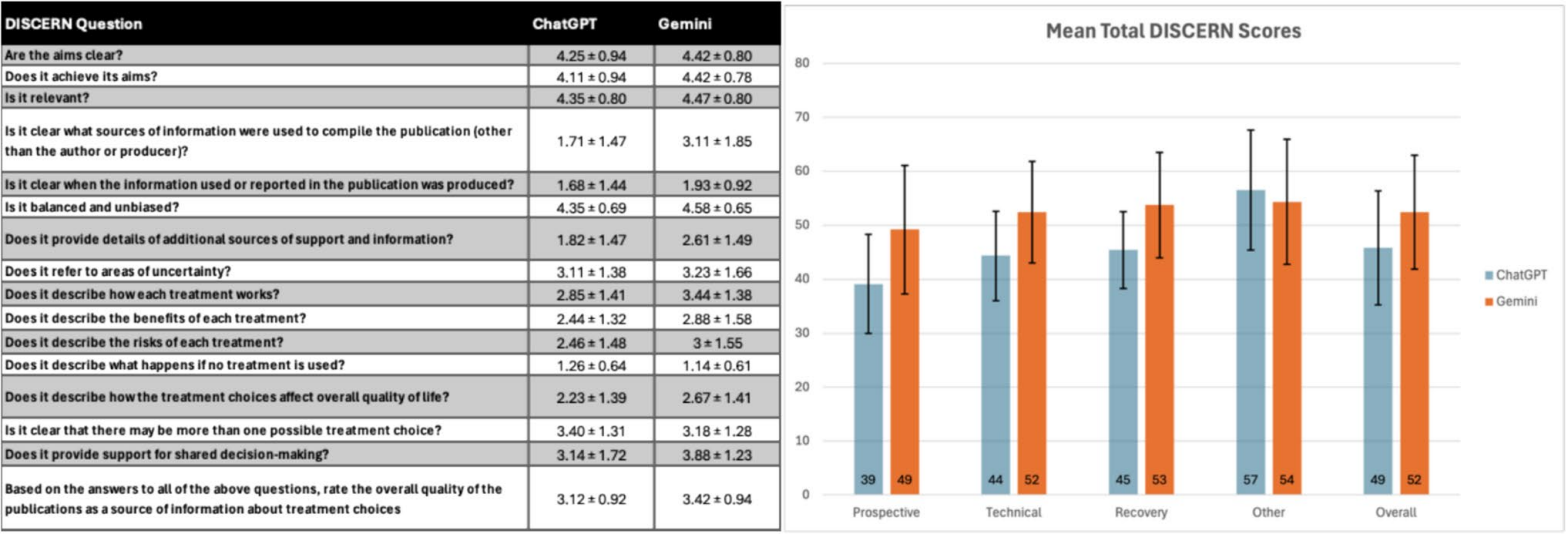
**A** Mean DISCERN scores (out of 5) with standard deviations for each response **B** Mean total DISCERN scores (out of 80) among each question category and overall. Numerical representation in the graph was rounded to the closest whole number for easier interpretation. Graphed value is still the true value. [56.5 represented numerically as 57, etc.…]

**Fig. 2:**
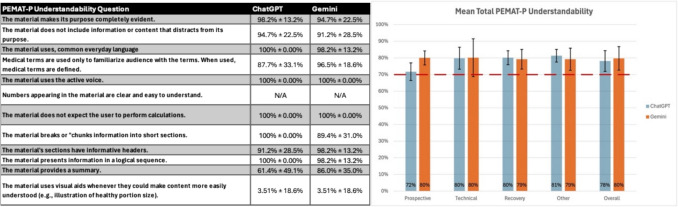
**A** Mean PEMAT-*P* Understandability scores with standard deviations for each response **B** Mean PEMAT-*P* Understandability scores among each question category and overall (70% minimum threshold for responses to be deemed “understandable”). Numerical representation in the graph was rounded to the closest whole number for easier interpretation. Graphed value is still the true value. [71.70% represented numerically as 72%, etc.…]

**Fig. 3:**
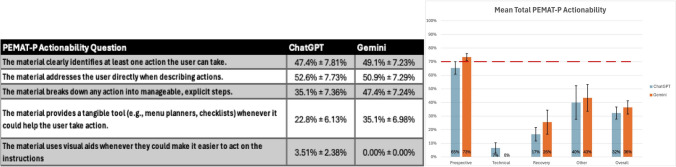
**A** Mean PEMAT-*P* Actionability scores with standard errors for each response **B** Mean PEMAT-*P* Actionability scores among each question category and overall (70% is the minimum threshold for responses to be deemed “actionable”). Numerical representation in the graph was rounded to the closest whole number for easier interpretation. The graphed value is still the true value. [65.40% represented numerically as 65%, etc.…]

**Conclusion:**

ChatGPT and Gemini deliver relevant and understandable information related to urologic telesurgery, with Gemini more consistently providing sources. However, neither chatbot reliably offers actionable responses, limiting their utility as a standalone gateway for patient decision-making.

**Supplementary Information:**

The online version contains supplementary material available at 10.1007/s11701-025-02871-8.

## Introduction

Traditionally, surgery has been performed with the surgeon and their team physically present in the operating room. Advancements in technology have led to the rise of laparoscopic surgery, followed by robotic-assisted procedures, and most recently, telesurgery. Telesurgery is defined as a surgeon performing robotic procedures using a robotic system that is controlled remotely, often in a different city or country [1]. With continuous advancements in communication networks and robotic technology, the increasing success of robotic-assisted surgery in urology has laid the foundation for urologic telesurgery. A 2021 study, lasting 8 months, measured the feasibility of urologic telesurgery, tracking 29 successful telesurgical radical nephrectomies with a median distance of 116 miles between the patient and surgeon [2]. This study, among others [3–5], shows the increasing success and new innovations of telesurgery, specifically within the field of urology.

As the popularity of telesurgery grows, patients are more likely to seek information due to the complexity of the concept. A 2018–2020 study found that 70–81% of Americans have looked up medical and health information online [6]. In line with the rising prevalence of AI chatbots, a cross-sectional study of 697 participants found that 78% of the respondents stated they would be willing to use ChatGPT for self-diagnosis [7]. ChatGPT and Google Gemini are two of the leading AI chatbot sources, with 600 and 350 million monthly active users, respectively [8].

With the very prevalent usage of these AI chatbots coupled with the growing use of telesurgical procedures, it is reasonable to assume that prospective patients may turn to AI chatbots for medical information. As a result, the precision and clarity of the medical information provided require rigorous assessments. There are many studies that have evaluated the medical information provided by AI chatbots in general and regarding more specific topics, such as head and neck cancer treatment [9–11]. However, there are no studies specifically evaluating the information provided by AI chatbots in reference to urologic telesurgery. This study aims to analyze and compare the quality of information provided by ChatGPT 4o and Google Gemini 2.5 regarding urologic telesurgery.

## Methods

### Search strategy and eligibility screening

A set of 19 questions related to urologic telesurgery was generated using general information from the American Urologic Association (AUA) and European Robotic Urology Section (ERUS). The questions were formatted to best mimic the language and vocabulary of an actual patient, and were then placed in one of 4 categories: Prospective, Technical, Recovery/Complications/Risks (labeled Recovery), and Other. “Prospective” questions were about patient eligibility and participation (e.g., How do I find a surgeon who performs urologic telesurgery?). “Technical questions” were about details of the procedure (e.g., What happens if there is a technical problem during my urologic telesurgery?). “Recovery” questions were about post-procedure workings (e.g., Is pain better managed after robotic urologic telesurgery compared to traditional surgery?). “Other” questions were considered miscellaneous and referred to questions that did not fit into any of the other categories (e.g., What if I change my mind about urologic telesurgery and want traditional surgery instead?). All 19 questions can be viewed in Supplementary File 1, provided after the conclusion. The questions were then typed directly into ChatGPT (OpenAI, San Francisco, CA) and Bard (Google, Mountain View, CA) [12, 13]. ChatGPT 4o and Gemini 2.5 were used. ChatGPT and Gemini are known to tailor their responses based on your conversation with them to provide more congruent answers. To avoid this confounding variable, for each question, a new conversation was generated to best replicate the experience a patient would have when they ask either of these chatbots a question. Responses were excluded if they were duplicates or were irrelevant to urologic telesurgery within reason. The remaining responses were considered for evaluation and scoring.

### Response review

Each individual response was evaluated by three medical students (TD, PG, MW). Responses were evaluated using the DISCERN and the Patient Education Materials Assessment Tool for Printed materials (PEMAT-*P*) tools. Both tools are validated for evaluating health information. DISCERN is a tool used to assess the quality of health information, with studies showing that both untrained individuals and healthcare professionals, alike, can effectively apply DISCERN to identify biases, gaps, and inaccuracies in medical information [14, 15]. The DISCERN survey includes 16 questions. The first 8 evaluate the reliability and sourcing of the information provided, while the next 7 focus on the clarity and completeness of details regarding treatment options. The final question asks the rater to give an overall assessment of the material’s quality. Each item is scored on a scale from 1 (definite no) to 5 (definite yes), with intermediate scores (2–4) reflecting partial fulfillment of the criteria. Individual question score interpretation is represented as follows: 4.01–5 = excellent, 3.01–4 = good, 2.01–3 = fair, 1.01–2 = poor, and 0–1 = very poor. Category scores are then summed, producing a total score that ranges from 16 to 80. Total scores are interpreted as follows: 68–80 = excellent quality, 55–67 = good quality, 42–54 = fair quality, 31–41 = poor quality, and below 30 = very poor quality.

While DISCERN evaluated the quality of the information provided, PEMAT-*P* is a standardized tool that focuses on the actionability and understandability of health information [16, 17]. PEMAT-*P* is composed of 24 questions, with the first 17 measuring understandability and the remaining 7 measuring actionability [16, 17]. Of the 24 questions, 7 were excluded as they were not applicable to the method we were using. Of the 7 excluded questions, 5 were excluded due to their emphasis on visual aids, 1 was excluded due to the limited need for calculations, and the final one was excluded as it referred to word counts typically associated with research papers and not AI generated responses.

### Interrater reliability

To measure agreeability between the three individual reviewers, interrater reliability (IRR) in Excel was calculated as a percentage through absolute agreement. The respective interrater reliabilities for the three surveys (DISCERN, PEMAT-*P* understandability, PEMAT-*P* Actionability) are listed in Table 1. Altman’s kappa benchmark scale was used to calculate the strength of agreement between the reviewers [18]. IRR scores for DISCERN were considered “Fair” and “Moderate” per Altman’s scale [18]. However, for PEMAT-*P* understandability/actionability, scores were considered “Very Good” [18]. We believe that the disparity in the score is due to the nature of the scoring. DISCERN used a Likert scale model of 1–5, whereas PEMAT used a binary 0/1. We believe that the introduction of partial credit in the DISCERN criteria led to decreased IRR scores.

**Table 1:** Interrater Reliability for the DISCERN, PEMAT-*P* understandability, and PEMAT-*P* actionability between each reviewer

Rater	DISCERN	PEMAT-*P* Understandability	PEMAT-*P* Actionability
TD-PG	0.39	0.95	0.93
TD-MW	0.42	0.91	0.93
PG-MW	0.57	0.94	0.93

## Results

### General characteristics

All 38 responses generated by both chatbots were deemed to be relevant to urologic telesurgery and were included. The average word count for responses was 353 and 579 words for ChatGPT 4o and Gemini 2.5, respectively. Gemini 2.5 more consistently provided sources for its answers (16/19 responses), while ChatGPT 4o was more limited (4/19 responses). Common sources included NIH, Johns Hopkins, and Mayo Clinic. Notably, neither chatbot utilized visual aids for, on average, only 3.51% of responses provided a visual aid for information clarity (Fig. 2). Statistical significance was calculated using paired T tests with a p value of < 0.05 indicating significance.

### Discern

Figure 1 represents the mean total DISCERN scores (out of 80) along with standard deviations for each category (Prospective, Technical, Recovery, Other, and Overall). DISCERN values are as follows for ChatGPT 4o versus Gemini 2.5, respectively: “Prospective” 39.1 versus 49.2; “Technical” 44.3 versus 52.4; “Recovery” 45.4 versus 53.7; “Other” 56.5 versus 54.3; “Overall” 45.8 versus 52.4 (Fig. 1). Statistically significant differences were noted between ChatGPT 4o and Gemini 2.5 in the “Prospective”, “Technical”, “Recovery”, and “Overall” categories with *p* values of 1.54 × 10^–7^, 5.95 × 10^–8^, 1.75 × 10^–10^, and 1.54 × 10^–15^, respectively. No statistical significance was noted in the “Other” category with a *p *value of 0.20. Gemini 2.5 overall outperformed ChatGPT 4o in all categories besides “Other”. DISCERN contained 16 questions, with the average score for each question represented in Fig. 1. ChatGPT 4o had 4 excellent, 4 good, 4 fair, and 4 poor average scores (Fig. 1). Gemini 2.5 had 4 excellent, 6 good, 4 fair, and 2 poor scores (Fig. 1). The lowest performing question for both chatbots was “Does it describe what happens if no treatment is used?” ChatGPT 4o scored an average of 1.26 on this question, while Gemini 2.5 scored a 1.14. ChatGPT 4o had a high score of 4.35 for two questions: “Is it relevant?” and “Is it balanced and unbiased?” Gemini’s highest scoring question was in “Is it balanced and unbiased?”, scoring 4.58. Regarding cumulative scores, ChatGPT 4o’s “Prospective” score was 39.1 (poor), and its “Other” score was 56.5 (good). Otherwise, the remaining 8 cumulative ChatGPT 4o and Gemini 2.5 DISCERN scores fell into the fair category.

### PEMAT-P understandability and actionability

Figure 2 represents the mean total PEMAT-*P* understandability scores for each category along with the mean score for each PEMAT-*P* understandability question. All categories met the 70% threshold to be considered understandable. PEMAT-*P* understandability values are as follows for ChatGPT 4o versus Gemini 2.5, respectively: “Prospective” 71.70% versus 80.0%; “Technical” 79.80% versus 80.10%; “Recovery” 80.10% versus 79.20%; “Other” 81.30% versus 79.20%; and “Overall” 78.10% versus 79.50% (Fig. 2). A statistically significant difference was only measured in the “Prospective” category with a *p* value of 3.6 × 10^–3^. No statistically significant difference was measured in the “Technical”, “Recovery”, “Other”, and “Overall” categories, with *p* values of 0.80, 0.62, 0.18, and 0.17, respectively. Gemini 2.5 performed better than ChatGPT 4o in the “Prospective” category, while the percentages in all other categories were separated by 1–2%. The lowest performing PEMAT-*P* question was “The material used visual aids whenever they could make content more easily understood” with both chatbots averaging a 3.51% for this question. Both chatbots scored 100% in many categories, including breaking up the information into sections, using concise language, and using an active voice, among others. The information provided by both chatbots was deemed relevant and understandable.

Figure 3 represents the mean total PEMAT-*P* actionability scores for each category along with the mean score for each PEMAT-*P* actionability question. Performance here was uniformly bad among the chatbots, with only Gemini 2.5’s score in the “Prospective” question category meeting the 70% threshold of being actionable. PEMAT-*P* Actionability values are as follows for ChatGPT 4o versus Gemini 2.5, respectively: “Prospective” 65.4% versus 73.4%; “Technical” 6.7% versus 0.0%; “Recovery” 16.7% versus 25.6%; “Other” 40.0% versus 43.4%; and “Overall” 32.3% versus 36.4% (Fig. 3). Statistically significant differences were noted in the “Technical” and “Recovery” categories, with both having *p* values of 0.045; however, scores in both these categories failed to meet the 70% threshold. No statistically significant differences were noted in the “Prospective”, “Other”, and “Overall” categories with *p* values of 0.083, 0.532, and 0.064, respectively. The lowest scoring PEMAT-*P* actionability question was “using visual aids” which they scored 3.51% and 0.00% for ChatGPT 4o and Gemini 2.5, respectively. While Gemini 2.5 had the highest score in “Prospective” questions, it also scored 0% in “Technical” questions. Limited scores in this category show that while the information provided by these chatbots has been deemed understandable and relevant due to previous scores, the actionability they provide remains limited.

## Discussion

With the increasing popularity of AI, its integration into medicine continues to grow. Currently, AI is being used in a variety of medical settings, including helping to take patient histories and assisting with administrative tasks [19, 20]. As physician use of AI continues to grow and evolve, it is safe to assume that patients will also turn to AI. As a result, it is important to evaluate the information provided by AI, specifically for medical questions in which inappropriate information can have direct consequences upon the patient. Other papers have compared information provided by ChatGPT and Gemini regarding specific procedures, including laparoscopic donor nephrectomies [21]. However, to our knowledge, this is the first study to analyze and compare the quality of information provided by ChatGPT 4o and Gemini 2.5 regarding urologic telesurgery. Urologic telesurgery is a field that is going to grow with continuous advancements in technology. The idea of a surgeon being able to perform on a patient while not being in the same room, let alone country, can be hard for patients to grasp. Hence, they may turn to AI chatbots for information.

In our analysis, most of the cumulative scores for DISCERN fell into the fair category (6/8) with ChatGPT 4o’s “Prospective” and “Other” questions landing in poor and good, respectively (Fig. 1). Statistically significant differences were present in the “Prospective”, “Technical”, “Recovery” and “Overall” categories, supporting the use of Gemini 2.5 over ChatGPT 4o. In addition to outperforming ChatGPT 4o overall, Gemini 2.5 performed notably better in providing sources for information. Regarding this, ChatGPT 4o scored a 1.71 on average, while Gemini 2.5 scored a 3.11. This is supported by a similar study looking at urogenital cancer information provided by an AI chatbot which had Gemini outperforming ChatGPT in the same category 4 to 1, respectively [22]. The specific model of Gemini and ChatGPT used in the prior study was not explicitly mentioned. However, while the sources provided differed between the two chatbots, the relevancy, clarity, and appropriate nature of the information provided were similar (Fig. 1).

All PEMAT-*P* mean understandability scores met the minimum 70% threshold for being deemed understandable (Fig. 2). The threshold of 70% was consistently met despite neither AI chatbot providing a visual aid more than a handful of times (3.51% for both) (Fig. 2). The lack of visual aids was counteracted by the consistent use of everyday language and breaking up of the material into sections for easier digestibility. Gemini 2.5 provided summaries for their answers more consistently, scoring 86% versus the 61.4% of ChatGPT 4o (Fig. 2). This could contribute to the notably larger average word count provided by Gemini 2.5 of 579 words versus ChatGPT 4o’s 353 words. While our study holds this notable word count difference, other studies have conflicting results, having ChatGPT having higher word counts than Gemini [22, 23]. One of these studies utilized ChatGPT v3.5, while the other utilized ChatGPT 4o. Overall, the only statistical significance regarding understandability was Gemini 2.5 outperforming ChatGPT 4o in the “Prospective” category.

While AI chatbot performance in for PEMAT-*P* understandability was sufficient, the opposite was found for PEMAT-*P* actionability. Only Gemini 2.5 in the “Prospective” question category met the 70% minimum threshold for responses deemed “actionable” (Fig. 3). Otherwise, all mean responses did not meet the minimum 70% threshold. Statistically significant differences were noted in “Technical” and “Recovery” categories; however, neither category met the minimum 70% threshold to be deemed actionable. Due to the variability of AI chatbots, there are conflicting results on actionability. Some studies show similar results in which both AI chatbots scored uniformly poorly on actionability [24, 25], while others have great results in actionability, meeting the 70% threshold [26, 27]. Some limitations within the actionability aspect of this study are that sometimes a question may not have a direct or clear actionable response. For example, if you were to ask an AI chatbot “what is urologic telesurgery”, it can provide a very relevant and understandable answer without necessarily providing any actionable information. However, all responses were still included in actionability grading as the chatbots would occasionally respond with phrases including “please consult with a urologist” or “check nearby hospitals and reference their websites”.

One potential application of AI chatbots based on our findings, along with other studies, would be the development of healthcare-specific AI chatbots to address common patient inquiries. Current AI chatbots use a general-purpose language model, and adjusting that to a healthcare-specific language model can be more appropriate for healthcare. Patients, rightfully so, will have questions about prospective procedures and appointments, and their best way to get accurate information is to call their provider. However, there are studies showing that unexpected telephone calls can result in a significant, unpredictable demand on workload for nurses and physicians [28, 29]. To help relieve the burden on the nursing staff and improve ease of access to patients, AI chatbots could be a solution. These chatbots could be programmed with accurate, up-to-date information tailored to the clinic’s services, policies, and patient population, allowing patients to obtain answers to frequently asked questions without calling the office, saving time for both the nurses and the patient. However, these chatbots must have strict safeguards that encourage the patient to call the office with any additional questions or confusion. While members of the public have trust in AI chatbots, there are existing risk concerns [30], which could be addressed with specific tailoring of these chatbots to their respective clinics.

There has been success in developing healthcare-specific large language models with Google’s Med-PaLM and the University of Florida’s GatorTron. Med-PaLM, now Med-PaLM 2, was designed for consumer health queries and became the first large language model to achieve a passing score on United States Medical Licensing Exam questions [31]. GatorTron is being developed by the University of Florida and was built on a large database of de-identified clinical data. The strengths of this model are in its ability to answer clinical questions and generate synthetic clinical text [32]. While both large language models have shown remarkable success in the clinical setting, their application is currently not available outside of research collaborations or institutional oversight. However, just the development of these models shows the practicality they may be able to have if accessible to the public. With appropriate safeguards and criteria meeting the clinical standard, if employed by clinics, healthcare-specific language models can be both beneficial to patients and their providers.

This study evaluated the information provided by ChatGPT 4o and Gemini 2.5; however, upon conclusion of this paper, ChatGPT 5 was released on August 7, 2025. Considering ChatGPT 5 being the “next step up from 4o,” another study should be performed comparing the medical information provided by GPT 5 against 4o and Gemini 2.5 to see if there is truly a significant difference.

## Limitations

While we believe this paper is exhaustive in comparing ChatGPT 4o and Gemini 2.5 in their respective abilities to provide accurate, understandable, and actionable information regarding urologic telesurgery, some limitations still apply. Responses were not blinded to reviewers, meaning they were able to see whether the responses were from ChatGPT or Gemini. However, we believe no explicit bias to be present here. As mentioned earlier, 19 questions were used, and while this list is detailed, it does not consider potential follow-up conversations. Instead, this study focused on the initial question a prospective patient may ask a chatbot, instead of all the potential follow-up questions, which leads to immense variability. Despite the introduction of ChatGPT 5, this study remains valid in nature as ChatGPT 4o is still available to be used due to overwhelming user preference.

## Conclusion

AI chatbots, ChatGPT 4o and Gemini 2.5, deliver relevant and understandable information related to urologic telesurgery. Of note, Gemini more consistently provides sources for its responses compared to ChatGPT, while neither chatbot consistently provided visual aids other than an occasional table. Additionally, neither chatbot reliably offers actionable responses. Their poor performance among PEMAT-*P* actionability criteria limits their utility as a standalone gateway for patient decision-making. This highlights the important role physicians have and will continue to have in patient care. Physicians should still be consulted as AI should not be used as a standalone measure for patients. Overall, the use of AI chatbots is effective to garner relevant information, but to acquire specific, actionable information, more detailed prompts/queries along with further discussion with their physician are required.

## Supplementary Information

Below is the link to the electronic supplementary material.

Supplementary file 1 (DOCX 124 kb)

## Data Availability

No datasets were generated or analyzed during the current study.
