# Supplementary material for: Quality assessment of patient-facing urologic telesurgery content using validated tools
Source: J Robot Surg. 2025 Oct 14;19(1):687. doi: 10.1007/s11701-025-02871-8 (PMC12521288; doi:10.1007/s11701-025-02871-8)
Supplement: Supplementary file 1 — (DOCX 124 kb) [file 11701_2025_2871_MOESM1_ESM.docx]

**Supplementary Files:**

**Supplementary File 1:** All 19 questions that were used to prompt ChatGPT 40 and Gemini 2.5 with regards to urologic telesurgery.
